# Natural Killer Cells in Systemic Autoinflammatory Diseases: A Focus on Systemic Juvenile Idiopathic Arthritis and Macrophage Activation Syndrome

**DOI:** 10.3389/fimmu.2019.03089

**Published:** 2020-01-15

**Authors:** Jessica Vandenhaute, Carine H. Wouters, Patrick Matthys

**Affiliations:** ^1^Laboratory of Immunobiology, Department Microbiology and Immunology, Rega Institute, KU Leuven – University of Leuven, Leuven, Belgium; ^2^UZ Leuven, Leuven, Belgium; ^3^European Reference Network for Rare Immunodeficiency, Autoinflammatory and Autoimmune Diseases (RITA) at University Hospital Leuven, Leuven, Belgium

**Keywords:** natural killer cell, sJIA, MAS, autoinflammation, immune-regulation

## Abstract

Natural killer (NK) cells are innate immune lymphocytes with potent cytolytic and immune-regulatory activities. NK cells are well-known for their ability to kill infected and malignant cells in a fast and non-specific way without prior sensitization. For this purpose, NK cells are equipped with a set of cytotoxic molecules such as perforin and apoptosis-inducing proteins. NK cells also have the capacity to produce large amounts of cytokines and chemokines that synergize with their cytotoxic function and that ensure interaction with other immune cells. A less known feature of NK cells is their capacity to kill non-infected autologous cells, such as immature dendritic cells and activated T cells and monocytes. Via the release of large amounts of TNF-α and IFN-γ, NK cells may contribute to disease pathology. Conversely they may exert a regulatory role through secretion of immuno-regulatory cytokines such as GM-CSF, IL-13, and IL-10. Thus, NK cells may be important target and effector cells in the pathogenesis of autoinflammatory diseases, in particular in those disorders associated with a cytokine storm or in conditions where immune cells are highly activated. Key examples of such diseases are systemic juvenile idiopathic arthritis (sJIA) and its well-associated complication, macrophage activation syndrome (MAS). sJIA is a chronic childhood immune disorder of unknown etiology, characterized by arthritis and systemic inflammation, including a daily spiking fever and evanescent rash. MAS is a potentially fatal complication of autoimmune and autoinflammatory diseases, and most prevalently associated with sJIA. MAS is considered as a subtype of hemophagocytic lymphohistiocytosis (HLH), a systemic hyperinflammatory disorder characterized by defective cytotoxic pathways of cytotoxic T and NK cells. In this review, we describe the established features of NK cells and provide the results of a literature survey on the reported NK cell abnormalities in monogenic and multifactorial autoinflammatory disorders. Finally, we discuss the role of NK cells in the pathogenesis of sJIA and MAS.

## Introduction

Natural killer (NK) cells are granular innate lymphocytes best known for their ability to kill infected and malignant cells in a fast and non-antigen specific manner. In humans, there is a general consensus for the existence of 2 NK cell subtypes based on the relative expression of CD56, a cell adhesion molecule, and CD16, also known as FcγRIII. In healthy donors, CD56^dim^CD16^+^ NK cells comprise around 90% of NK cells in peripheral blood and are mainly cytotoxic. The other 10% are CD56^bright^CD16^dim/−^ NK cells and produce greater amounts of cytokines than CD56^dim^CD16^+^ NK cells (1). NK cells originate from hematopoietic stem cells that are differentiated to common lymphoid precursor cells and in a traditional view the CD56^bright^ NK cells are considered as precursor cells of the CD56^dim^ subset. However, this concept has been challenged as there is evidence that the two subsets can be seen as separate populations with different origin and characteristics (2).

**Figure 1:**
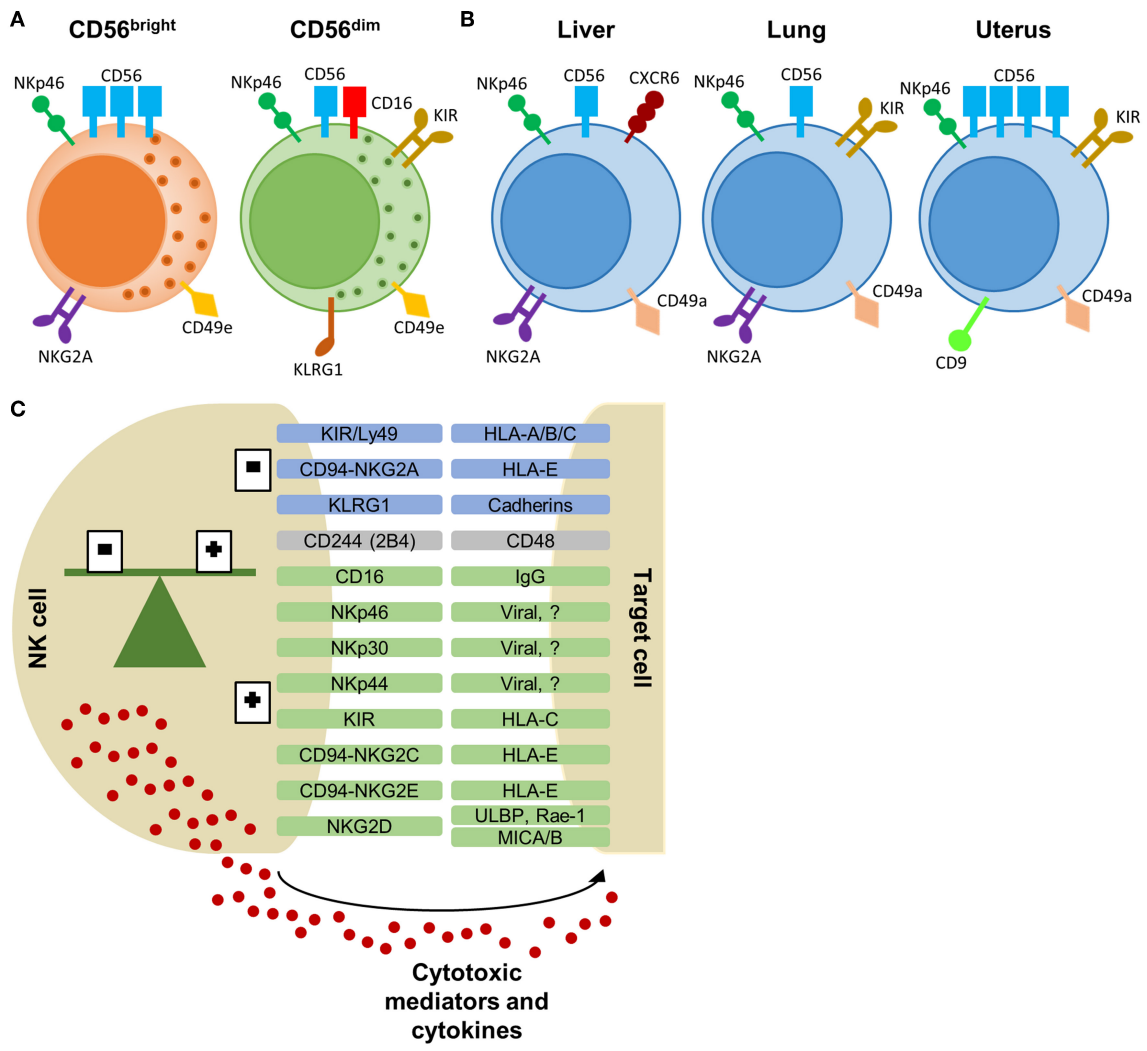
NK cell subtypes and receptor-ligand interaction between NK cell and target cell. **(A)** CD56^bright^ and CD56^dim^ NK cells, primarily found in the peripheral blood, with a selection of characteristic markers. **(B)** Tissue-resident NK cells from the liver, lung, and uterus express overlapping markers with peripheral NK cells. Each tissue-specific NK cell subtype displays a characteristic phenotype which allows differentiation from peripheral NK cells. **(C)** Selection of NK cell receptors and their corresponding ligands on target cells. Inhibitory receptors are in blue, activating receptors are in green and receptors with dual function are in gray. The balance of inhibitory and activating signals define the activity or tolerance of NK cells. Activated NK cells will release cytotoxic proteins and cytokines to eliminate the target cells. KIR, killer-immunoglobulin receptor; HLA, human leukocyte antigen; KLRG1, killer cell lectin-like receptor G1; ULBP, UL16 binding protein; Rae, Retinoic acid early inducible; MIC, MHC class I polypeptide-related sequence.

More recently, NK cells have been classified as a group 1 innate lymphoid cell (ILC), comprising conventional (c)NK cells and ILC1s (3). ILC1s are tissue-resident, also known as tissue-resident (tr)NK cells, and are virtually found in all organs, including liver, lung, and uterus, whereas cNK cells are circulating via the blood stream (3). Although ILC1s and NK cells have multiple common features, they are functionally and phenotypically different. cNK cells have a stronger cytotoxic potential with higher expression of perforin compared to ILC1s. In contrast, ILC1s have only weak cytotoxic function with low perforin levels, but produce high levels of cytokines like interferon (IFN)-γ, tumor necrosis factor (TNF)-α, and granulocyte-macrophage colony-stimulating factor (GM-CSF) (4, 5). In addition, trNK cells have been shown to confer adaptive features, i.e., hapten- and virus-induced memory (6–8). Besides these differences in function, ILC1s and NK cells have a distinct phenotype. Both cell types express characteristic NK cell markers, i.e., CD56, NKp46, CD122 (IL-2 receptor β), and activating receptor NKG2D, whereas other markers can be used to discriminate subsets of NK cells (9, 10). The subtypes of NK cells and their characteristics have been reviewed extensively elsewhere (2, 9), of which we provide a short overview here. In the peripheral blood, CD56^bright^ NK cells can be distinguished from CD56^dim^ NK cells via the higher expression of CD56 and the absence of CD16 (Figure 1A). In addition, CD56^bright^ NK cells are characterized by the expression of NKG2A, CXCR3, and CCR7. In contrast, CD56^dim^ NK cells express CXCR1, CXCR2, S1P5, and have a higher expression of killer immunoglobulin-like receptors (KIR), and maturation markers KLRG1 and CD57 (9, 11). Furthermore, peripheral blood NK cells can be distinguished from tissue-specific NK cells via the expression of CD49e (9, 12). Specialized tissue-specific NK cells have been described in liver, lung, and spleen (Figure 1B) (4, 12–17). In general, trNK cells largely resemble CD56^bright^ NK cells with a tissue-specific expression of adhesion and tissue-retention markers, such as CD69 and chemokine-receptors CXCR6 and CCR5 (4, 9, 16). In addition, trNK cells lack the expression of CD49e, KIR, CD16, and maturation marker CD57 (4, 9, 12). Unlike for murine trNK cells, the expression of CD49a is not specific for human trNK cells (4, 16, 18). A more distinct subtype of NK cells can be found in the uterus (Figure 1B). Uterine NK cells have a CD56 “superbright” phenotype, express CD9 and KIR, and show more characteristic tissue-resident features by the expression of CD69 and CD49a (9, 19). Uterine NK cells produce growth-promoting factors, have an important placental vascular remodeling function during pregnancy and are thought to provide memory for this vascular remodeling in subsequent pregnancies (19, 20).

NK cells can exert their cytotoxic function either via perforin-release or via the engagement of death receptors TNF-related apoptosis-inducing ligand (TRAIL) or Fas ligand (21). NK cells also have an important immune-modulatory function by the release of cytokines and chemokines (22). The most prominent cytokines produced by NK cells are IFN-γ and TNF-α. NK cells also secrete immuno-regulatory cytokines such as GM-CSF, IL-13, and IL-10 (23–25). In addition, NK cells also produce a variety of chemokines, including CXCL8 (IL-8), CCL2 [monocyte chemoattractant protein (MCP)-1], CCL3 [macrophage inflammatory protein (MIP)-1α], CCL4 (MIP-1β), CCL5 (RANTES), and CXCL10 [IFN-inducible protein (IP)-10] (25, 26). As part of the innate immune system, NK cells do not need prior sensitization to exert these functions, though activation with cytokines, i.e., type I IFN, IL-2, IL-12, IL-15, and IL-18, greatly enhances their activity (1). IL-18 is an important cytokine for stimulation of NK cell cytotoxicity and IFN-γ production by NK cells (27). Also IFN-γ has been shown to drive NK cell function. The importance of the IL-18/IFN-γ axis in NK cell function is further highlighted by the impaired NK cell function in IL-18- and IFN-γ-deficient mice (28, 29). Next to the innate function of NK cells, recent reports have demonstrated adaptive features of NK cells, and more specifically trNK cells, with a hapten-, virus-, and cytokine-induced memory function, thereby enabling NK cells to respond with higher efficacy and enhanced cytotoxic and cytokine-producing activity upon restimulation (6–8, 30).

NK cell activity is tightly regulated via a large repertoire of inhibitory and activating NK cell receptors to avoid off-target effects (31). NK cell receptors can be classified in different subsets based on their receptor function or by the nature of their ligands (Figure 1C). Signaling by most inhibitory and activating receptors is mediated via conserved sequences in the cytoplasmic region of the NK cell receptor. Inhibitory receptors share an immunoreceptor tyrosine-based inhibitory signaling motif (ITIM), whereas activating receptors have an immunoreceptor tyrosine-based activation motif (ITAM) (31). The CD94/NKG2-family, including NKG2A, and the murine Ly49 or human KIR-family recognize MHC class I ligands. Both inhibitory and activating receptors are found in these families. The activating receptor NKG2D recognizes MHC class I-related proteins, including retinoic acid early inducible (Rae)-1 in mice and UL16 binding protein (ULBP) and MHC class I polypeptide-related sequence (MIC)A/B in humans. CD48 is the ligand for 2B4 (CD244) and is expressed on all hematopoietic cells. 2B4 can act as an inhibitory or activating receptor depending on the expressed isoform. For many NK cell receptors (e.g., KLRG1 and NKp46), their function or ligand-specificity remains unknown. The family of natural cytotoxicity receptors, i.e., NKp46, NKp44, and NKp30, are specifically expressed by NK cells. These receptors can activate the NK cell via recognition of pathogen-derived proteins and self-ligands (1, 31–33).

During maturation, NK cells undergo an educational process that allows them to discriminate between healthy cells and target cells via two mechanisms. Firstly, downregulation of MHC class I molecules by transformed and infected cells results in a lack of inhibitory signals (missing-self recognition). On the other hand, stressed cells can upregulate expression of stimulatory ligands, overruling the signals of inhibitory NK cell receptors (induced-self recognition). Both mechanisms result in a shifted balance toward activation of the NK cells and target cell lysis (30, 34). The latter mechanisms allows NK cells to regulate activated cells. It has been shown that NK cell cytotoxicity has an important role in homeostasis and induction or down-regulation of the cellular immune response (35, 36). On the one hand, NK cells can stimulate the adaptive immune response via IFN-γ and chemokine production. On the other hand, NK cells can kill autologous activated T cells, dendritic cells, and monocytes (1, 37–40), which could be important in terminating the immune response when necessary. Together with the secretion of anti-inflammatory cytokine IL-10, NK cells are now considered as complex immune-regulatory cells in striking contrast to their pathogenic effector role via the release of IFN-γ and the induction of tissue damage via cytolysis (1, 41, 42).

Autoinflammatory syndromes comprise a group of rare, genetically diverse, but clinically distinct pathologies characterized by recurrent fever, rash, and lymphadenopathy, accompanied by cutaneous, mucosal, serosal, and osteoarticular inflammation. Autoinflammatory diseases are associated with constitutive inflammasome activation and a dysregulation of the innate immunity. The classification, etiology, and pathogenesis of the diverse monogenic and multifactorial autoinflammatory diseases has been extensively reviewed elsewhere (43–48). The role of NK cells in autoinflammatory diseases remains largely unknown. In this review, we will give an overview of the pathogenic and regulatory features of NK cells in the context of autoinflammation.

## The Inflammasome and Natural Killer Cell Activation

Activation of innate immune pathways is a hallmark of autoinflammation. The innate immune system protects the body from pathogens in a fast and non-specific manner, in contrast to the antigen-specific adaptive immune system. Innate immune cells, such as macrophages, dendritic cells and neutrophils, detect pathogens or cell damage via pattern recognition receptors (PRRs) which recognize pathogen- or damage- associated molecular patterns (PAMPs or DAMPs). Activation of PRRs initiates the expression of chemokines, cytokines, enzymes, and adhesion molecules and the recruitment of leukocytes. The PRR family includes multiple receptors, amongst which are TLRs, NOD-like receptors (NLRs) and RIG-I-like receptors (RLRs). The TLR family exists of 11 receptors recognizing various extracellular or endolysosomal bacterial and viral PAMPs. These receptors initiate a signaling cascade via the Toll/IL1 receptor (TIR) domain and the MyD88 adaptor, except for TLR3, which signals via the TIR-domain-containing adapter-inducing interferon-β-dependent pathway. The RLR family mediates the expression of pro-inflammatory cytokines via the recognition of pathogenic nucleic acids in the cytoplasm. Finally, the NLR family consist of intracellular PRR proteins, which upon triggering activate signaling through the NF-κB pathway (NOD1/2) or through inflammasome complexes (NLRP1, NLRP3, and NLRC4) (49, 50). Multiple autoinflammatory disorders are associated with mutations in inflammasome-related genes (49, 51), resulting in constitutive activation of the inflammasome and cleavage of pro-IL-1β and pro-IL-18 via caspase-1 into active IL-1β and IL-18. The latter have been identified as main drivers of disease in autoinflammatory disorders (49, 52, 53).

More recently, inflammasome activation and the release of IL-18 has been shown to be a critical checkpoint in the activation of NK cells and the induction of memory NK cells in the liver (54, 55). Activation of the NLRC4 inflammasome (via intracellular bacteria) primed NK cells to efficiently kill infected hepatocytes in a perforin-dependent manner and thereby enhancing control of the infection (56). Alternatively, carcinoma-derived proteins were found to activate the NLRP3 inflammasome, which resulted in FasL-mediated NK cell cytotoxicity against metastatic tumor cells and effective tumor suppression after IL-18 activation of NK cells (57). Also, the NLRP3 inflammasome in tissue-resident macrophages was involved in the induction of hapten-dependent memory function of NK cells and was necessary to establish contact hypersensitivity against monobenzone (58). Inflammasome-derived IL-18 efficiently primed NK cells resulting in higher cytotoxic potential (56–58).

These reports can be of great value for understanding the role of NK cells in autoinflammatory syndromes with constitutive activation of the inflammasome.

## Natural Killer Cells in Monogenic Autoinflammatory Diseases

Monogenic autoinflammatory syndromes are associated with genetic defects in a single gene. Reports on the functionality or numbers of NK cells in this group are scarce or even non-existing for the majority of monogenic autoinflammatory diseases (Table 1), most likely due to the low prevalence of most of the monogenic autoinflammatory diseases.

**Table 1:** NK cells in monogenic autoinflammatory disorders.

**Monogenic autoinflammatory disorder (****44**, **45**, **47****)**		**Gene**	**Reports on NK cells**
FMF	Familial mediterranean fever	*MEFV*	↑ NK cell numbers (59) ↓ CD16^+^ NK cells (60) KIR2DS2 association (61)
TRAPS	TNF receptor-associated periodic syndrome	*TNFRSF1A*	*De novo* missense variant (62) ↓ NK cell numbers (63)
CAPS	Cryopyrin-associated periodic syndrome		/
-FCAS	Familial cold autoinflammatory syndrome	*NLRP3*	/
-MWS	Muckle-Wells syndrome	*NLRP3*	/
-NOMID	Neonatal-onset multisystemic inflammatory disorder	*NLRP3*	/
MKD/HIDS	Mevalonate kinase deficiency/hyperimmuno-globulinemia D syndrome	*MVK*	/
DIRA	Deficit of IL-1 receptor antagonist	*IL1RN*	/
PAPA	Pyogenic arthritis pyoderma gangrenosum and acne syndrome	*PSTPIP1*	/
FCAS2	Familial cold autoinflammatory syndrome 2	*NLRP12*	/
Majeed syndrome		*LPIN2*	/
Blau syndrome		*NOD2/CARD15*	/
DITRA	Deficiency of IL-36 receptor antagonist	*IL36RN*	/
JMP	Joint contractures, muscle atrophy, and panniculitis-induced lipodystrophy syndrome	*PSMB8*	/
CANDLE	Chronic atypical neutrophilic dermatosis with lipodystrophy and elevated temperature syndrome	*PSMB8*	/
NNS	Nakajo-Nishimura syndrome	*PSMB8*	/
CAMPS	CARD-14-mediated pustular psoriasis	*CARD14*	/
NALP12-associated periodic fever		*NALP12*	/
APLAID	Autoinflammation and phospholipase Cγ2-associated antibody deficiency and immune dysregulation	*PLCγ2*	↓ NK cell numbers ↓ NK cell degranulation ↓ NKG2D and 2B4 signaling (64)

*↑, increased; ↓, decreased compared to control group; /, no reports available. References are indicated in the table*.

Conflicting reports exist on the number of NK cells in patients with familial mediterranean fever (FMF, mutation in *MEFV*, resulting in abnormal regulation of IL-1β activation). One study reported higher NK cell numbers in patients with FMF (59), whereas a recent study demonstrated decreased CD16^+^ NK cells as compared to the control group (60). The presence of activating KIR2DS2 was found to be associated with FMF (61).

In a patient with TNF receptor-associated periodic syndrome (TRAPS, mutation in *TNFRSF1A*, resulting in abnormal TNF-receptor function) a *de novo* missense variant in the *TNFRSF1A* gene was found. Interestingly, the mosaic variant allele was detected specifically in B cells, NK cells, and neutrophils, but not monocytes and T cells, potentially indicating an important role for NK cells along neutrophils in the TRAPS pathogenesis (62). Another study reported decreased numbers of NK cells in patients with TRAPS as compared to healthy controls (63).

Ombrello et al. described a defective NK cell function and signaling in patients with autoinflammation and phospholipase Cγ2-associated antibody deficiency and immune dysregulation (APLAID, mutation in *PLC*γ*2*, resulting in abnormal B cell function) syndrome. In these patients, NK cell numbers and the CD107a degranulation were decreased and a reduced signaling activity was observed downstream of the receptors NKG2D and 2B4 (64).

We found no reports on NK cells in patients with cryopyrin-associated periodic syndrome (CAPS), including familial cold autoinflammatory syndrome (FCAS), Muckle-Wells syndrome, and neonatal-onset multisystemic inflammatory disorder (NOMID), mevalonate kinase deficiency/hyperimmuno-globulinemia D syndrome (MKD/HIDS), deficit of IL-1 receptor antagonist (DIRA), pyogenic arthritis pyoderma gangrenosum and acne syndrome (PAPA), familial cold autoinflammatory syndrome 2 (FCAS2) Majeed syndrome, Blau syndrome, deficiency of IL-36 receptor antagonist (DITRA), joint contractures, muscle atrophy and panniculitis-induced lipodystrophy (JMP) syndrome, chronic atypical neutrophilic dermatosis with lipodystrophy and elevated temperature (CANDLE) syndrome, Nakajo-Nishimura syndrome (NNS), CARD-14-mediated pustular psoriasis (CAMPS), and NALP12-associated periodic fever.

## Natural Killer Cells in Multifactorial Autoinflammatory Diseases

Next to monogenic autoinflammatory disease, another group of autoinflammatory diseases present with an uncertain genetic etiology and are considered to have a polygenic or multifactorial cause (43, 44). Also for multifactorial autoinflammatory disorders, NK cell studies remain scarce (Table 2). More reports are available on the more prevalent multifactorial autoinflammatory disorders, including Behçet's disease, Crohn's disease and Still's disease. The latter will be discussed in the next section.

**Table 2:** NK cells in multifactorial autoinflammatory disorders.

**Multifactorial autoinflammatory disorder (****43**, **44****)**		**Reports on NK cells**
PFAPA	Periodic fever, aphthous stomatitis, pharyngitis, and adenopathy syndrome	= numbers of CD57^+^ NK cells (65)
Schnitzler's syndrome		↑/ = percentage NK cells (66)
SAPHO	Synovitis acne pustulosis hyperostosis osteitis syndrome	↓ NK cell numbers (67)
CRMO	Chronic recurrent multifocal osteomyelitis	/
Sweet's disease		/
Behçet's disease		↓(68, 69) / = (70) / ↑ (71) NK cell numbers ↓/ = NK cell cytotoxicity (69, 71–74) ↑/ = CD107a degranulation (68, 70, 72) ↓ perforin and granzyme B expression (69) KIR association (75–77) ↑ IFN-γ production (68, 72, 78, 79)
Crohn's disease and ulcerative colitis (UC)		↓ (80) / = (81) NK cell numbers ↓ (81, 82) / = (83, 84) NK cell activity ↓ NKG2D^+^ NK cells in lamina propria (85) ↑ NKp46^+^ (Crohn) /NKp44^+^ (UC) NK cell in mucosa (86) ↑ risk: KIR2DL2, KIR2DS2, KIR2DL5 and KIR2DS1 (UC) (87) ↓ risk: KIR2DS3 (Crohn's) (88)

*↑, increased; ↓, decreased; =, equal compared to control group; /, no reports available. References are indicated in the table*.

In patients with periodic fever, aphthous stomatitis, pharyngitis and adenopathy (PFAPA) syndrome, staining of tonsillar tissue revealed normal numbers of CD57^+^ NK cells (65). In a small study of patients with Schnitzler syndrome, one out of the two included patients presented with a highly increased percentage of NK cells (66). A relatively large study of 19 patients with synovitis acne pustulosis hyperostosis osteitis (SAPHO) syndrome reported reduced numbers of NK cells and increased numbers of Th17 cells in SAPHO patients compared to controls, suggesting an imbalance of NK cells and inflammatory Th17 cells may underlie the immune inflammation in patients with SAPHO (67). We found no reports on NK cells in patients with chronic recurrent multifocal osteomyelitis (CRMO) and Sweet's disease.

Reports on peripheral NK cells function in Crohn's disease and ulcerative colitis (UC) demonstrated normal to lower NK cell numbers and NK cell cytotoxic activity, during active disease and remission (80–83). Although NK cell activity was elevated upon intravenous IFN-γ infusion in a clinical trial with a limited number of patients with Crohn's disease, no clinical improvement was reported, suggesting a limited role for IFN-γ-induced NK cell activity in the pathogenesis of Crohn's disease (84). Nevertheless, the association of KIR genes in the susceptibility for Crohn's disease and UC suggests involvement of NK cells in disease development. KIR2DL2, KIR2DS2, KIR2DL5, and KIR2DS1 were found to confer a higher UC susceptibility, whereas a negative association was found between KIR2DS3 and Crohn's disease risk (87, 88). More recent reports have focused on NK cells in tissues in contrast to the peripheral circulating NK cells, resulting in the identification of organ-specific and tissue-resident NK cells. In the gut, a novel subset of mucosal NK cells has been identified. These mucosal NK cells are characterized by the expression of the transcription factor retinoic acid-related orphan receptor C (RORC), CD127 (IL-7Rα), and the production of IL-22 (89). In patients with Crohn's disease, NKp46^+^ mucosal NK cells were increased which produced IFN-γ upon IL-23 stimulation. In contrast, in patients with UC, NKp44^+^ IL-22-producing mucosal NK cells were elevated (86, 90). The potential pathogenic or protective role of these NK cells in chronic gut inflammation is still unknown (90). A potential pathogenic role for NK cells in Crohn's disease was established via the successful treatment of patients with Crohn's disease with anti-NKG2D antibody (91). Expression of NKG2D, predominantly on T cells, and its ligands, MHC class I polypeptide-related sequence (MIC)A/B and UL16 binding protein (ULBP), were highly elevated on lesions of patients with active Crohn's disease and UC (92). Upregulation of these ligands, can activate NK cells to kill these activated or stressed autologous cells, indicating a potential pathogenic pathway (92, 93). Nevertheless, in a dextran sulfate sodium (DSS)-induced mouse model of UC, NKG2D^+^ NK cells were found to be decreased in the lamina propria during active disease, proposing a regulatory role for NK cells in this mouse model of UC (85). In general, NK cells are thought to have a dual role in gut inflammation with a pathogenic role in Crohn's disease and UC via cytotoxic activity and cytokine secretion, and a protective role against the development of cancer (90, 93).

Also in patients with Behçet's disease, there are several conflicting reports on NK cell numbers, phenotype and activity. NK cell numbers were reported to be lower (68), normal (70), or even increased (71) in patients with Behçet's disease compared to controls. One study described the decreased presence of NK cells in bronchoalveolar lavage (BAL) fluid in patients with Behçet's disease with pulmonary manifestations (69). Phenotypically, NK cells of patients with Behçet's disease showed a slightly skewed NK cell receptor repertoire with increased NKG2D, decreased perforin and granzyme B expression, and abnormal KIR expression (69, 70, 75, 76). Association of KIR2DL3 gene expression suggests that NK cell activity is involved in the pathogenesis of Behçet's disease (77). Nevertheless, other reports state a normal NK cell phenotype with normal NKG2D (72), NKG2A, NKp30 or NKp46 expression (70) or normal perforin and granzyme B expression (68). Polymorphisms in *CD94/NKG2A, CD94/NKG2C, ERAP1, KLRC4, CCR1*, and *STAT4* were associated with Behçet's disease (94–96). The exact effect of these genetical variations on Behçet's disease remains unknown. Since the genes were either directly or indirectly linked to NK cell activity, it was hypothesized that potential defects in NK cells would result in diminished NK cell function and persistent inflammation following a pathogenic trigger (96, 97). Indeed, a normal to decreased cytotoxic activity was observed in multiple studies in patients with Behçet's disease (69, 71–74). In contrast, a normal to high degranulatory capacity was observed in patients with Behçet's disease after tumor cell stimulation (68, 70, 72). Interestingly, patients with active Behçet's disease showed high IFN-γ production by NK cells, which was thought to contribute to disease relapse (68, 72, 78, 79). In accordance, patients with inactive Behçet's disease had an impaired IL-12-induced STAT4 phosphorylation, associated with lower IFN-γ production. NK cells from inactive Behçet's patients were also able to suppress IFN-γ production by CD4^+^ T cells, suggesting a regulatory role for NK cells in disease remission (74).

## Natural Killer Cells in sJIA and MAS

Systemic juvenile idiopathic arthritis (sJIA), or Still's disease, is a severe immune-inflammatory childhood disorder, classified as one of the subtypes of juvenile idiopathic arthritis (JIA). According to ILAR classification, sJIA is diagnosed in the presence of arthritis in one or more joints with or preceded by quotidian fever of at least 2 weeks duration, and accompanied by evanescent erythematous rash, enlargement of lymph nodes, liver, and/or spleen or serositis (98). In adults, a comparable disorder to sJIA can occur and is referred to as Adult-onset Still's disease (AOSD) (99). sJIA is associated with the potentially life-threatening complication macrophage activation syndrome (MAS). Around 10% of sJIA patients develop MAS, with subclinical MAS reported in up to 50% of the patients (100, 101).

MAS is a potentially life-threatening hyperinflammatory syndrome associated with excessive activation and proliferation of macrophages and CD8^+^ T cells leading to an overwhelming cytokine storm and hemophagocytosis (102). MAS closely resembles hemophagocytic lymphohistiocytosis (HLH) and is therefore classified as a form of secondary HLH (sHLH). Primary or familial HLH and secondary forms of HLH, including MAS, share most clinical and biological manifestations. Both conditions are characterized by severe inflammation with high morbidity and increased mortality risk. Patients present with a persistent high fever, lymphadenopathy, and hepatosplenomegaly. In addition, liver dysfunction and central nervous system involvement are frequently observed. Similar to familial or genetic HLH, MAS is characterized by a decrease of several blood cell lines, leading to anemia, thrombocytopenia, and leukopenia. CRP levels are increased. Further, patients present with increased liver enzymes, including AST, ALT, and LDH, increased bilirubin levels, hypoalbuminemia, hyponatremia, and hypertriglyceridemia. Patients also present with increased D-dimers and decreased fibrinogen levels, resulting in a decreased ESR. A severe coagulopathy may ensue and cause multi-organ failure and death in 8–22% of patients (103, 104). An important marker of MAS is the highly increased levels of ferritin. At last, hemophagocytic macrophages are found in bone marrow and tissue biopsies (102, 105–107).

Genetic or fHLH is due to mutations in genes associated with the cytotoxic pathway of cytotoxic T cells (CTL) and natural killer (NK) cells. Conversely, sHLH is not associated with monogenic defects in the cytotoxic pathway and can occur as a complication of infections, malignancies, immunosuppressive therapy, and autoimmune and autoinflammatory diseases (108). In the context of autoimmune and autoinflammatory diseases, sHLH is referred to as MAS. MAS can occur in the context of rheumatic diseases, including systemic lupus erythematosus, Kawasaki disease, AOSD and rheumatoid arthritis, and in monogenic autoinflammatory diseases, amongst which CAPS and FMF (105, 109). Nevertheless, MAS is most frequently reported in patients with sJIA. It has been suggested that NK cell dysfunction represents a common pathway in patients with sJIA, MAS complicating sJIA and HLH (35). In the following sections, we will review NK cell-linked genetical abnormalities and NK cell function in patients with sJIA and MAS complicating sJIA (Table 3).

**Table 3:** NK cells in sJIA, MAS, and fHLH.

	**sJIA/AOSD**	MAS/sHLH*	**fHLH**
**GENETIC ABNORMALITIES IN CYTOTOXICITY-RELATED GENES**			
*PRF1*		(110–113)	fHLH-type2
*UNC13D*	(114)	(112, 113, 115–117)	fHLH-type3
*STX11*		(113)	fHLH-type4
*STXBP2*		(112, 117)	fHLH-type5
*LYST*		(111, 117)	Chédiak-Higashi syndrome
*Rab27A*		(118)	Griscelli syndrome
*AP3B1*			Hermansky-Pudlak syndrome type 2
*SH2D1A*		(113)	XLP-1
*BIRC4*			XLP-2
*other*		(111, 117)	
**NUMBERS OF NK CELLS**			
Total	↓ (119–124) = (125–129)	↓ / = (35, 130)	= (35)
CD56^dim^	↓ (119, 122) / = (125)		
CD56^bright^	↓ (121, 128) / = (124, 125)		
**NK CELL CYTOTOXICITY AND RELATED PROTEINS**			
Cytotoxicity	↓ (110, 120, 121, 123, 128, 129, 131) = (125)	↓ (130)	↓ (132)
CD107a	↓ (121) / = (125)	= (133)	↓(133)
Perforin	↓ (110, 120, 121, 123, 131) = (125)	↓ (110, 130) = (130)	↓ (134) = (35)
Granzyme A	= (125)		
Granzyme B	↓ (120, 123) / ↑ (125)		
Granzyme K	↓ (125)		
**CYTOKINE PRODUCTION BY NK CELLS**			
IFN-γ	↓° (121, 125) ↑^#^ (120, 124)		↑ (135)
TNF-α	↓^#^ (120)		

XLP, X-linked lymphoproliferative syndrome;*, MAS comprises sJIA-associated MAS and sHLH comprises virus-associated HLH and late-onset onset HLH; ↑, increased compared to control group; ↓, decreased compared to control group; =, equal with control group; °, after stimulation with IL-18; #, after stimulation with PMA and ionomycin. References are indicated in the table.

### Genetical Abnormalities Linked to NK Cells in sJIA and MAS

The pathogenesis of MAS has been deducted from HLH due to its clinical and biological similarity. In fHLH, mutations in genes regulating granule-dependent cytotoxicity cause defective cytolysis by NK cells and CTLs underlying the excessive inflammation (136). The exact mechanism that links defective cytolysis with excessive and ongoing inflammation remains elusive. It is hypothesized that NK cells and CTLs in patients with fHLH/MAS fail to eliminate infected cells, which leads to persistent antigenic stimulation. This ongoing stimulation enhances immune activation, excessive proliferation of T cells and production of cytokines, resulting in a self-amplifying inflammatory activity (35, 137). Another hypothesis suggests that defective cytotoxic NK cells and CTLs fail to induce apoptosis to remove activated antigen-presenting cells (APC) and T cells. This failure to terminate the immune response leads to a persistent inflammatory response (35, 42, 137).

fHLH has been linked to mutations in 9 genes which are inherited in an autosomal recessive or X-linked manner. Each of the underlying mutations affects a different protein involved in the granule-mediated cytolytic pathway and is therefore associated with a different subtype of HLH or an HLH-related immunodeficiency syndrome (108, 137). Although the subtypes all share a similar clinical phenotype, disease severity and onset of disease is different according to the affected cytotoxicity-related protein (138, 139). Mutations in *PRF1*, encoding cytotoxic effector protein perforin, is associated with subtype fHLH-2 and early onset of disease (108, 138). Defective perforin expression results in failure to induce apoptosis in the targeted cell, contributing to a prolonged synapse time and an impaired cytotoxic activity (140). Mutations in *UNC13D* are associated with fHLH-type 3. The gene encodes Munc13-4, a protein with a non-redundant role in the priming of the cytolytic granules and consequently fusion of the granule with the plasma membrane. Mutations in *STX11*, encoding Syntaxin-11, and *STXBP2*, encoding Munc18-2, are associated with fHLH subtype 4 and 5, respectively. Syntaxin-11 and Munc18-2 interact and regulate fusion of granules with the plasma membrane (108).

HLH-related primary immunodeficiency syndromes are associated with mutations in genes involved in the cytotoxic pathway, including *LYST* (encoding for Lyst, involved in Chédiak-Higashi syndrome), *RAB27A* (Rab27a, Griscelli syndrome 2), *AP3B1* (AP3, Hermansky-Pudlak syndrome type 2), *SH2D1A* (SAP, X-linked lymphoproliferative syndrome (XLP)-1), and *BIRC4* (XIAP, XLP-2). These mutations are linked to more general defects in trafficking and exocytosis of lysosomes, resulting in impaired functions in multiple cell types, including neurons, melanocytes, platelets, granulocytes, and lymphocytes. Of note, mutations in *UNC13D, STX11*, and *STXBP2* also influence exocytosis processes in platelets and neutrophils, in addition to cytolytic degranulation (108, 136).

With regard to MAS, no loss-of-function mutations in fHLH-associated genes have been described. Nevertheless, numerous studies have documented polymorphisms in genes associated with granule-mediated cytotoxicity in MAS patients. Vastert et al. reported heterozygous missense mutations in *PRF1* in 20% of sJIA patients with a history of MAS, compared to only 9.8% of sJIA patients without MAS (110). Zhang et al. reported polymorphisms in the *UNC13D* gene in 11 out of 18 patients with MAS complicating sJIA. Two patients presented with bi-allelic sequence variants, 9 of the 16 other patients had a common pattern of sequence variants comprising 12 single nucleotide polymorphisms (SNPs). The genetic variations were highly associated with MAS-complicated sJIA (57%) compared to uncomplicated sJIA (8.2%) and healthy controls (12%) (115). SNPs in *UNC13D* were also found in 2 sJIA-associated MAS patients in another cohort (116). Remarkably, a single-patient study described heterozygous mutations in the *UNC13D* gene associated with reduced NK cell cytotoxic function in sJIA (114). More recently, Schulert et al. described a novel heterozygous intronic variant of *UNC13D* associated with impaired NK cell degranulation in a patient with sJIA and recurrent MAS episodes (141). In contrast, another study in 133 sJIA patients found no association between SNPs in *PRF1, UNC13D, GZMB*, and *Rab27a* and sJIA (142). Whole exome sequencing in 14 MAS-complicated sJIA patients revealed heterozygous protein-altering variants in fHLH-associated genes (*LYST, UNC13D*, and *STXBP2*) in 35.7% of patients compared to only 13.7% in uncomplicated sJIA patients. The functional significance of these variants was not investigated. Next to these known genes, heterozygous protein-altering variants and SNPs were found in a number of genes indirectly associated with cytotoxicity, including *SLAC2B, XIRP2, MICAL2, CADPS2, ARHGAP21, CCDC141, FAM160A2*, and *LRGUK*, through an effect on microtubule reorganization and vesicle transport (117).

Also in virus-associated HLH, heterozygous mutations in *LYST* and *PRF1* have been described in patients with fatal sHLH following H1N1 influenza infection. No protein-altering variants were found in *UNC13D, STX11, STXBP2*, or *Rab27a*. The study also identified other protein-altering variants in genes associated with cytoskeleton stabilization (*XIRP2*) and microtubule structure (*LRGUK*), which were described as MAS-associated genes by Kaufman et al. (111, 117). Also NK cell receptor-related genetic variations, especially KIR polymorphisms were found in EBV-associated HLH, with higher susceptibility in carriers of KIR2DS5 or KIR3DS1 (143).

The identification of heterozygous variants in genes directly and indirectly linked to cytotoxicity pathways in MAS and infection-associated HLH blurs its distinction from late-onset fHLH. A heterozygous *Rab27a* mutation was identified in 2 adolescents with HLH (118). A large study on adult HLH patients found hypomorphic mutations in *PRF1, UNC13D*, and *STXBP2* in 14% of patients correlating with a later-onset of disease (112). Another study identified mutations in HLH-related genes in 18 out of a total of 252 adolescent and adult patients. The majority of these patients (50%) presented with mutations in *PRF1*, followed by 38.8% of patients with *STX11* missense mutations. *SH2D1A* mutations were found in 2 patients of whom 1 patient also had a *PRF1* mutation, and 1 patient had a *UNC13D* missense mutation. No variations were found in *STXBP2* or *BIRC4*. The biallelic and monoallelic mutations corresponded with a very low to low, respectively, NK cell activity compared to controls. The authors suggest that these “milder” genetic mutations, in a functionally unimportant region of the protein, may be a predisposing factor to late-onset HLH (113). In contrast, high-throughput sequencing of genetically undiagnosed late-onset HLH patients found no enriched mono-allelic variations compared to the healthy population. The authors suggest caution with the interpretation of causality and identification of genetic variants in disease (144). Indeed, other factors than genetics can trigger the development of HLH. The presentation of HLH as a genetic or secondary form has recently shifted toward considering it as a threshold-disease. A combination of predisposing factors (i.e., genetics, underlying diseases, immunosuppression, infection,…) are accumulated until a certain threshold is reached, leading to uncontrolled inflammation. This model comprises a wide spectrum of HLH, from fHLH, to MAS, and sHLH (108, 145, 146).

Although no clear monogenic defects in cytotoxicity-related genes can be associated with MAS complicating sJIA, the genetic variations observed in some studies can reflect subpopulations of patients and further highlight the role of NK cell dysfunction in the development of MAS. In addition, these observations suggest the involvement of inflammation-driven factors underlying cytotoxic defects of NK cells in sJIA and MAS.

### NK Cell Dysfunction in sJIA

The role of NK cells in the pathogenesis of sJIA remains incompletely understood. Contradictory results concerning numbers or activity of NK cells in sJIA patients have been reported, which at least partially can be explained by the small numbers of patients included in these studies together with a high heterogeneity of the disease course of sJIA patients (147).

Most studies report low to mildly decreased numbers of NK cells in the PBMC fraction of sJIA and AOSD patients (119–124). Nevertheless, other research groups described normal numbers of NK cells (125–129). Correspondingly, the proportion of NK cell subsets was also altered in sJIA patients. Some studies found decreased CD56^dim^ NK cells (119, 122), whereas others reported decreased CD56^bright^ NK cells (121, 128). In contrast, equal numbers of CD56^dim^ and CD56^bright^ NK cells have been reported when comparing sJIA or AOSD patients and healthy controls (124, 125). Phenotypically, only subtle alterations in the expression of NK cell receptors are observed in sJIA patients (120, 121, 125).

NK cell dysfunction in sJIA is thought to be one of the contributory features for its strong association with MAS. Nevertheless, whether an intrinsic cytotoxic defect can be found in patients with sJIA and AOSD remains undecided. Although, multiple studies reported decreased NK cell cytotoxicity in PBMCs from sJIA patients (110, 120, 121, 123, 128, 129), normal NK cell cytotoxicity was detected when the cytotoxicity was calculated relative to the (decreased) numbers of NK cells (125). Lower expression of cytotoxic proteins (i.e., perforin and granzyme B) was associated with this decrease in NK cell function (110, 120, 121, 123, 148). Interestingly, autologous stem cell transplantation in sJIA patients restored this decreased expression of perforin (148). Contradictory, RNA sequencing of NK cells of sJIA patients revealed normal transcriptional expression of perforin and granzyme A and B. In the same study, protein levels of these cytotoxic proteins in NK cells were found to be normal-to-increased, further supporting the reported intact NK cell cytotoxicity. Furthermore, Put et al. described decreased expression of granzyme K in CD56^bright^ NK cells at protein and transcriptional level (125). The expression of granzyme K by CD56^bright^ NK cells has been linked to the killing of autologous activated T cells in patients with multiple sclerosis (149). This regulatory aspect of NK cells has not been investigated so far in patients with sJIA. However, by using a novel mouse model for sJIA (150), we recently found a cytotoxic defect in NK cells of the diseased animals and further provided evidence that NK cells play a regulatory role in the development of the disease via a NKG2D-dependent control of inflammatory monocytes (151).

Since the functional, phenotypical and transcriptional data have not allowed to identify an intrinsic cytotoxic defect in NK cells of patients with sJIA, a transient NK cell dysfunction induced by the continuous inflammatory environment has been proposed (125, 152). This effect is mainly thought to be driven by the excessive levels of IL-18 found in patients with sJIA. Although IL-18 is a stimulatory cytokine for cytotoxicity and cytokine production by NK cells, it failed to induce a cytotoxic response, an increased perforin expression and degranulation by NK cells (121, 153). More surprisingly, stimulation with IL-18 did not elicit production of IFN-γ by sJIA NK cells, which was caused by a reduced phosphorylation downstream of the IL-18 receptor β (121, 125). Of note, NK cells of sJIA and AOSD patients were still capable of IFN-γ and TNF-α production after triggering with other stimulatory factors (120, 124, 125). This discordance between the high plasma levels of IL-18 and relatively low IFN-γ expression are most likely reflecting a state of hyporesponsiveness toward IL-18 in patients with active sJIA. At the time of MAS, patients seem to recover IL-18 responsiveness resulting in high plasma levels of IFN-γ in contrast to sJIA (125, 152, 154, 155). Next to IL-18, the high levels of IL-6 during sJIA are also associated with decreased cytotoxicity with decreased perforin and granzyme B expression, which could be recovered by tocilizumab (131). The NK cell dysfunction seen in patients with sJIA could be a consequence of the systemic inflammation, but whether this is a predictive factor of MAS development in all of these patients remains unclear.

### NK Cell Deficits in MAS

Functional deficits of NK cells are generally accepted to be part of the pathogenesis of MAS (as discussed above). Indeed, Grom et al. reported decreased cytotoxicity of NK cells in MAS-complicated sJIA patients. Interestingly, two patterns were observed, mimicking secondary HLH and genetic HLH. Firstly, some patients presented with low NK cell activity associated with drastically decreased NK cell numbers but mildly increased perforin expression. On the other hand, very low NK cell cytotoxicity was associated with mildly decreased NK cell numbers but highly decreased expression of perforin (130). Contradictory, NK cell degranulation assays demonstrated abnormal NK cell activity in only 22% of patients with secondary HLH, including patients with MAS (133). Phenotypical studies of NK cells of MAS patients are scarce. Increased NKG2A and decreased NKG2D expression on NK cells were reported in a study on sHLH patients, including MAS patients (156).

## Conclusion

The role of NK cells in autoinflammatory disorders remains elusive. In general, a defective NK cell function and diminished NK cell numbers are observed. Nevertheless, for many monogenic and multifactorial autoinflammatory diseases (i.e., Crohn's disease, UC and Behçet's disease) no decisive conclusion can be made, due to the low number of studies or the contradictory results.

The role of NK cells in sJIA and MAS has been studied extensively. Nevertheless, there are no robust genetical or transcriptional defects observed in the cytotoxic pathways of NK cells in patients with sJIA and MAS complicating sJIA. Although, functional defects in cytotoxicity and/or cytokine production have frequently been observed in these patients, a number of conflicting data have been reported for patients with sJIA, which probably reflects the disease heterogeneity in sJIA (122, 157, 158). In MAS, the data are conclusive for functional deficits of NK cells. Since inflammasome activation and the release of IL-18 efficiently activates NK cell function, one would assume highly active NK cells in patients with sJIA and MAS complicating sJIA, both characterized by extremely high IL-18 levels. Nevertheless, an inflammation-induced NK cell exhaustion, mediated via the constitutively high levels of cytokines (IL-18 and IL-6), has been hypothesized to be part of the pathogenesis of sJIA and MAS. The resulting cytokine-induced NK cell dysfunction, leads to failure to terminate the immune response and thus an ongoing inflammation (35, 42, 137).

## Author Contributions

JV wrote the first draft of the review. JV, CW, and PM wrote and revised the final version of the review. All authors contributed to manuscript revision, read and approved the submitted version.

### Conflict of Interest

The authors declare that the research was conducted in the absence of any commercial or financial relationships that could be construed as a potential conflict of interest.
